# Soft-Tissue Simulation for Computational Planning of Orthognathic Surgery

**DOI:** 10.3390/jpm11100982

**Published:** 2021-09-29

**Authors:** Patricia Alcañiz, Jesús Pérez, Alessandro Gutiérrez, Héctor Barreiro, Ángel Villalobos, David Miraut, Carlos Illana, Jorge Guiñales, Miguel A. Otaduy

**Affiliations:** 1 Department of Computer Science, Universidad Rey Juan Carlos, 28933 Madrid, Spain; jesus.perez@urjc.es (J.P.); hector.barreiro@urjc.es (H.B.); miguel.otaduy@urjc.es (M.A.O.); 2 GMV Innovating Solutions, 28760 Madrid, Spain; avillalobos@gmv.com (Á.V.); dmiraut@gmv.com (D.M.); cillana@gmv.com (C.I.); 3 Fundación Para la Investigación Biomédica del Hospital Universitario La Paz, 28029 Madrid, Spain; alessandro.gutierrez.venturini@idipaz.es; 4 Hospital Universitario La Paz, 28046 Madrid, Spain; diazdecevallos@gmail.com

**Keywords:** soft-tissue simulation, finite-element model, surgical planning, orthognathic surgery

## Abstract

Simulation technologies offer interesting opportunities for computer planning of orthognathic surgery. However, the methods used to date require tedious set up of simulation meshes based on patient imaging data, and they rely on complex simulation models that require long computations. In this work, we propose a modeling and simulation methodology that addresses model set up and runtime simulation in a holistic manner. We pay special attention to modeling the coupling of rigid-bone and soft-tissue components of the facial model, such that the resulting model is computationally simple yet accurate. The proposed simulation methodology has been evaluated on a cohort of 10 patients of orthognathic surgery, comparing quantitatively simulation results to post-operative scans. The results suggest that the proposed simulation methods admit the use of coarse simulation meshes, with planning computation times of less than 10 seconds in most cases, and with clinically viable accuracy.

## 1. Introduction

Orthognathic surgery is performed on patients who suffer from dentomaxillofacial disharmony. Deformities of maxillofacial bones may prevent proper functioning of these bones, which are key for chewing, breathing and speaking [1]. They may also cause important functional problems such as sleep apnea, malocclusion problems, or lack of skeletal harmony. In addition to functional problems, facial aesthetic appearance is also often a motivation for orthognathic surgery, in combination with orthodontic treatment [2]. The aim of orthognathic surgery is to correct the maxillofacial deformities and dental occlusion, by cutting and repositioning maxillary and/or mandibular bones [3,4].

In the last decades, thanks to technological and scientific progress, virtual surgical planning has evolved tremendously, enabling pre-operative planning of interventions such as orthognathic surgery. Planning of orthognathic surgery is carried out in close collaboration between orthodontists and maxillofacial surgeons, and leverages existing 3D surgical planning solutions [5,6,7].

However, current solutions for orthognathic surgical planning suffer a trade-off between accuracy and speed. Several commercial software solutions, such as Dolphin (Dolphin Imaging & Management Solutions, CA, USA) or Maxilim (Medicim NV, Mechelen, Belgium), offer interactive planning methods, albeit with crude approximations of the biomechanical response. They approximate the response of soft tissue after bone repositioning as a simple geometric transformation, without accurate mechanical modeling.

In contrast, multiple research works have studied computational models of soft tissue for accurate prediction of the surgical outcome [6,8,9,10,11,12,13]. Most of these works rely on complex finite-element models (FEM), and they need up to several minutes to estimate the deformations due to simulated surgical interventions [8,12,14].

### 1.1. Contributions

This study proposes a soft-tissue simulation methodology that achieves high accuracy thanks to an FEM foundation, but it also provides high computational efficiency for a semi-interactive planning experience. A key novelty in this proposal is to approach two major tasks of soft-tissue modeling, namely mesh preparation and mathematical modeling, in a cross-informed manner. Previous methods pay a performance penalty due to poor treatment of the boundary conditions between soft tissue and bones. These boundary conditions are expressed with fine geometric detail that leads to high-resolution discretizations, and are solved using computationally costly methods. Instead, we model boundary conditions in a manner that allows the use of simple and fast numerical solvers, and we design procedures for mesh preparation that simplify the definition of boundary conditions between soft tissue and bones. As a result, simulation models bear a complexity that is well under the standards of previous methods, with the accompanying benefit on simulation performance, but without compromising accuracy.

**Figure 1:**
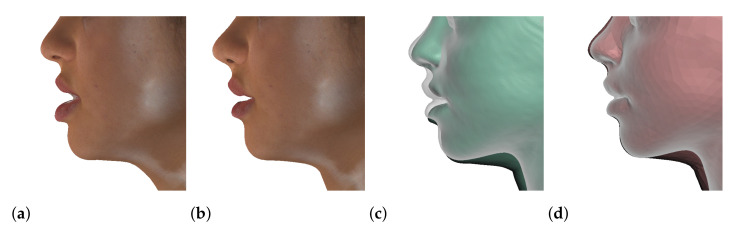
These images depict one of the patients (M8) of the test cohort. The first two images compare a pre-operative 3D scan (**a**) and a deformation of this scan produced by our planning simulation (**b**). Next, the pre-operative scan, in green, is overlaid with a post-operative scan, in grey, to highlight the effect of the actual surgery (**c**). The final image compares our simulation result, in brown, to the post-operative scan (**d**). In the planning simulation, 86% of the deformed surface attains an error of less than 3 mm with respect to the post-operative scan, and the error is concentrated at the neck. Moreover, this high accuracy is achieved with an FEM simulation that takes less than 4 s to compute.

We have tested the proposed simulation methodology on a cohort of 10 orthognathic surgery patients. For all these patients, we obtain both pre- and post-operative data, which allows us to simulate surgical planning while replicating true interventions, and validating the predicted result with respect to the post-operative data. We achieve consistently a simulation performance of a couple seconds, with accuracy comparable to that of high-resolution simulations. Figure 1 shows one example result, and compares it to pre- and post-operative scans.

### 1.2. Background

Before describing in detail our methodology, we review previous work on simulation and planning procedures for orthognathic surgery. The amount of works that document the use of simulation in a clinical context is immense, and we discuss only a few with particular contributions.

#### 1.2.1. Classification of Previous Work

Bobek et al. [15] proposed the use of an intraoral fiducial marker for accurate prediction of lip deformation, which could be integrated with other methodologies and increase accuracy. The study of Lee et al. [16] is particularly notorious due to the size of the cohort, with 204 patients. And Xia et al. [17] explain the complete planning procedure to a clinical audience. In [18], the authors summarize the work carried out at their lab for over 15 years, discussing possible extensions of soft-tissue simulation to the evaluation under motion or ageing of the face.

The use of soft-tissue FEM simulation for predicting orthognathic surgery was demonstrated at least 25 years ago [19,20]. Since then, many works have shown the application of FEM soft-tissue simulation for planning of orthognathic surgery, covering a broad range of interventions. Some of the interesting additions to baseline FEM models include the use of muscle fibers [21], accurate volume preservation [22], or efficient collision handling based on distance fields [23]. We also list here works that developed a full planning software based on FEM simulation [24,25,26,27]. However, these works do not validate their methods clinically.

The relevance of orthognathic surgery has favored the existence of multiple commercial software solutions for planning. Some works evaluate the features of these solutions, Dolphin (Dolphin Imaging & Mangement Solutions, CA, USA) [28], SurgiCase CMF (Materialise, Leuven, Belgium) [29], or 3dMD Vultus (3dMD Inc., Atlanta, GA, USA) [30]. The recent review by Olivetti et al. [7] analyzes multiple modern software solutions.

In the remainder of this section, we pay detailed attention to works that validate soft-tissue simulation by comparing simulation results to actual clinical interventions. We discuss these works according to the main components of our methodology.

#### 1.2.2. Simulation Meshes

The first step in the preparation of the simulation scene is to define simulation meshes for the soft tissue and the bones. Most approaches use a pre-operative CBCT-scan of the patient as input, and the different relevant anatomical elements are segmented and then meshed. Segmentation may be carried out following manual or semi-automatic approaches. Some works complement the CBCT-scan with a 3D scan of the patient’s face [8].

A different approach to the preparation of the simulation meshes is to consider only the patient’s face as input [8,9,11,12]. In some of these cases, a precomputed template is adapted to the patient’s specific anatomy [8,12].

Our methods use as input a CBCT-scan of the head and a 3D scan of the face. We segment the patient’s preoperative CBCT image and consider the entire head at a volumetric level (with the bones embedded in the soft tissues of the oral cavity), and not just the skin around the face. Moreover, we separate the lips, which move following maxilla and mandible separately and therefore are crucial for a correct result [9,12,13,31]. We complement the input data further with a dental scan, which we register to the CBCT image, for higher accuracy of the simulation at the teeth.

Simulations are typically executed on volumetric meshes, but some works use also surface meshes [10]. The volumetric mesh can be either tetrahedral [6,9,11,13,14,32] or hexahedral [12,31]. The number of elements and nodes is normally around some thousands [6,12,31] but can go up to a million [14].

In this proposal, we use volumetric meshes of tetrahedra created using TetGen (v. 1.5, WIAS, Berlin, Germany [33]) (same as [9]). Keeping the simulation meshes at a moderate resolution is key for computational efficiency of planning simulation. However, it is non-trivial to achieve the desired accuracy with low-resolution meshes. We succeed to do this by carefully modeling boundary conditions, which allows the use of an efficient solver.

#### 1.2.3. Soft-Tissue Model

Most of the previous works use FEM models, either self-developed [8,10,11,13], or built in commercial software such as Ansys (Ansys Inc, Canonsburg, PA, USA) [12,14,32]. Some works use other models, such as the mass-spring model [9] or the mass-tensor model [11,34]. Mollemans [6] compared these three models.

The materials used in simulation vary between homogeneous linear elastic materials [6,10,32], heterogeneous linear materials that separate muscle and adipose tissue [11,12,13,31], or nonlinear materials [6,35]. Some studies take into account viscoelasticity [14] and different parameters values [13] to reach optimum simulation results.

In this proposal, we use a self-developed FEM implementation with a nonlinear Neo-Hookean material. Our experiments suggest that nonlinearity and heterogeneity are not crucial for a correct prediction of the planning output on interventions applied to the bones, but they might be necessary if detailed interventions are executed, e.g., on the lips.

One crucial aspect for predictive planning is correct modeling of boundary conditions. The usual approach is to move the bones and let the soft tissue deform according to their displacement, as in real surgery. This allows clinicians to explore the result under different transformations to bones. Soft tissue can be classified into different groups, depending on its interaction with bones. The most accurate models consider tissue groups such as fixed, free, bonded or sliding [11,31]. Only a few works take into account the sliding effect between the lips and the teeth [8,11,32]. The displacement applied to the bones may depend on the test case, such as only the maxilla [13,14], only the mandible [35], or both [6,8,9,10,11,36].

We consider all types of boundary conditions between soft tissue and bones, i.e., fixed, free, bonded or sliding, as discussed above. We let the clinician apply cuts to the bones and transform bone fragments independently. A key aspect of our approach is to handle efficiently the bonded interaction between soft tissue and bone fragments, by splitting the initial coupling into face groups. This feature is implemented efficiently in our self-developed soft-tissue solver.

#### 1.2.4. Performance and Validation

In previous FEM-based methods, the simulation of orthognathic procedures required in the order of a few minutes [8,12,14]. Mollemans [6] executed simulations in just half a minute, but using highly simplified geometry and without considering the separation of the lips. Semi-interactivity, i.e., computation time of just a few seconds, was reached only using models based on the mass-spring or mass-tensor methods [11,34].

Most of the studies have performed clinical validations on small cohorts of patients (fewer than 10 patients). However, some studies have used considerably larger cohorts (e.g., 25 patients in [10] and 40 patients in [8]). Our study falls in the mid range, with a cohort of 10 patients.

The validation may be either qualitative or quantitative. Qualitative validation uses questionnaires answered by surgeons [6,8,9,11,12]. Quantitative validation performs error measurement between the simulated output and actual post-operative results. Several studies report low errors, between 1 and 3 mm, which guarantee practical applicability [6,8,9,10,11,12,13]. We also follow quantitative validation, by measuring error with respect to post-operative results.

Our work brings a notable contribution in its combination of computational efficiency and thoroughness of the validation. We present modeling and simulation methods that enable accurate predictions in just a few seconds. Moreover, we have validated these methods on a cohort of 10 patients, which cover a diverse set of clinical cases.

## 2. Materials and Methods

As discussed in the introduction, soft-tissue modeling entails two tasks, which are typically addressed separately: mathematical modeling of soft-tissue deformations and mesh preparation. By approaching these two tasks in a cross-informed manner, we minimize the requirements on the soft-tissue models, and therefore we maximize run-time simulation efficiency. In this section, we describe the methods we follow for mathematical modeling and mesh preparation, highlighting how these two tasks interplay.

The section starts with the description of the soft-tissue model, which adopts state-of-the-art finite-element methods (FEM). The section continues with a brief description of our bone model, based on rigid bodies. Next, it describes how we tackle boundary conditions in a compact and efficient manner. We find that a compact and modular approach to specify boundary conditions is a key element for simulation efficiency, often underestimated. Modular handling of boundary conditions simplifies the preparation of simulation meshes as well as run-time input of user-defined manipulations. Based on the compact protocol for the definition of boundary conditions, the section continues with the preparation of the simulation meshes and the configuration of boundary conditions, both at initialization and at run-time. Finally, this section discusses the presentation of the planning results with high-quality visualization.

### 2.1. Mathematical Modeling of Soft Tissue

We model as soft tissue all relevant soft anatomical elements of the face, such as lips, gums, internal muscle of the face, the top area of the neck, and the skin. We consider all these elements as one elastic continuum, which is connected to bone elements as we will discuss later. Considering all soft tissue as a continuum may be a crude approximation for certain analysis, but it appears sufficient for orthognathic surgery planning, as suggested by our experiments. Previous works in the literature differ widely in their choices of material models and material parameters. Despite a predominance of linear elasticity [10,13,14,31,32], some consider nonlinear materials [6,12]. The value of Young modulus is as far as 3 kPa [31] or 1 MPa [14]. After some tests, we opted for a (nonlinear) Neo-Hookean material with a Young modulus of 100 kPa and Poisson’s ratio of 0.47. The facial tissue is admittedly heterogeneous, but our results suggest that our approximation is sufficient for the application at hand.

Since we adopt state-of-the-art FEM for soft-tissue modeling, we keep this description short. We formulate soft-tissue simulation as a numerical optimization problem, and we outline the degrees of freedom of this problem, as well as the objective function or energy that is minimized. The interested reader may follow the bibliography for more information on material models [37], the finite-element discretization [38], and computer implementation [39].

Let us define the undeformed and deformed positions of points within the elastic soft tissue as *X* and *x*, respectively. Deformation can be characterized by the deformation gradient $F=\frac{\partial x}{\partial X}$. Based on the deformation gradient, a constitutive law defines the elastic energy density of the material. In our simulation model, we adopt a Neo-Hookean material model, with energy density

(1) $$\Psi \left(F\right)=\frac{\mu }{2}\;\left(\mathrm{trace}\left(F^{T}\;F\right)-3\right)-\mu \;\log\left(\det(F)\right)+\frac{\lambda }{2}\;{\left(\log\left(\det(F)\right)\right)}^{2}.$$

The Lamé constants μ and λ are set based on the Young modulus and Poisson ratio of the material.

In a computer model, the deformation field *x* is discretized at a set of nodes, and we denote as x a vector of degrees of freedom (DoFs) that concatenates the positions of all nodes. In the remainder, we use boldface font to denote vectors and matrices that assemble quantities over all discrete elements. The deformation field is interpolated in the continuum using shape functions as x=N(X)x. In our simulations, we use tetrahedral elements with linear shape functions. The deformation gradient can be obtained by differentiating the deformation field as $F=\frac{\partial N}{\partial X}\;x$.

The FEM solution to the elasticity problem yields the nodal deformations x which minimize the total energy $V_{\mathrm{soft}}\left(x\right)$ of the soft tissue. This energy is composed of the volume-integral of the elastic energy density Ψ(F) and the work of external forces $f_{\mathrm{ext}}$. In practice, the integral is computed using quadrature, as the weighted sum of energy values computed at discrete integration points, with weights $\left\{w_{i}\right\}$. With our choice of linear finite elements, one integration point per element is sufficient, and its weight is just the volume of the corresponding tetrahedron. The total energy can be summarized as:

(2) $$V_{\mathrm{soft}}=\int_{\Omega }\Psi \left(F\right)\;d\Omega -f_{\mathrm{ext}}^{T}\;x\approx \sum_{i}w_{i}\;\Psi \left(F_{i}\right)-f_{\mathrm{ext}}^{T}\;x.$$

Note that we have decided to ignore gravity in our analysis. As the soft tissue is already pre-loaded with gravity forces in its initial configuration, adding gravity would produce wrong deformations. A more accurate approach would be to estimate the undeformed shape of the soft tissue such that, with the inclusion of gravity, it is at rest at the initial configuration. However, our experiments suggest that this is not necessary, and dropping gravity is a reasonable approximation.

In an unconstrained setting, the deformation of the soft tissue is obtained by finding the roots of a force equilibrium problem $f_{\mathrm{int}}+f_{\mathrm{ext}}=0$, where the internal forces are computed as the negative gradient of the elastic energy, $f_{\mathrm{int}}=-\sum_{i}w_{i}\;{\frac{\partial N}{\partial X}}^{T}\;{\frac{\partial \Psi }{\partial F}}^{T}$. In this expression, $\frac{\partial \Psi }{\partial F}$ denotes the First Piola-Kirchhoff stress, and the shape function derivatives $\frac{\partial N}{\partial X}$ transform the stress into nodal forces. However, we propose to model boundary conditions, in particular the interaction of soft-tissue and bone elements, using constraints. Then, the elastic deformation problem needs to be reformulated as a constrained optimization, as detailed later in Section 2.4.

### 2.2. Mathematical Modeling of Bones

We choose to model bones as rigid bodies, and our results seem to confirm that the deformation of bony structures is irrelevant for planning of orthognathic procedures. Based on this design choice, we start by discussing how we augment the DoFs of the system x with the DoFs of rigid bones. The configuration of a rigid bone can be parameterized by the position of its center of mass $x_{b}$ and a rotation matrix $R_{b}$. Then, a point with rest position *X* is transformed to a world position $x=R_{b}\;X+x_{b}$. At each iteration of the simulation, we reparameterize the rotation using an incremental rotation in the tangent space [40]. Describing this incremental rotation using an axis angle $\theta_{b}$, the world position is reformulated as $x=R_{b}\;X-\left(R_{b}\;X\right)\times \theta_{b}+x_{b}$. This expression defines the instantaneous kinematics of a bone; therefore, for each bone, we augment the system DoFs x with the center of mass $x_{b}$ and the incremental rotation $\theta_{b}$.

As bones are rigid, they do not contribute any elastic energy. Moreover, due to our gravity-free approximation, they do not contribute any internal energy to (2). However, bones affect the formulation of the overall problem through boundary conditions, i.e., constraints, which we discuss next.

### 2.3. Mathematical Modeling of Boundary Conditions

Our simulations support different constraints. We focus our attention on contact constraints and tissue coupling. Carefully modeling these constraints allows us to formulate soft-tissue simulation as a computationally efficient problem.

#### 2.3.1. Sliding Contact

The simulation must support sliding contact between soft and/or rigid surfaces. Mathematically, contact can be modeled as constraints that prevent interpenetration. However, instead of hard constraints, for contact we use soft constraints, i.e., we model elastic energies that penalize interpenetration. This choice is robust when contact occurs between volumetric objects, for which penetration depth can be robustly computed; and this is the case for all the anatomical elements involved in the orthognathic scene.

We start by defining a non-penetration constraint for each individual contact. Given two contact points $x_{p}$ and $x_{q}$, with collision normal *u*, we formulate a non-penetration constraint as $u^{T}\;\left(x_{p}-x_{q}\right)=0$. We can express all contact points in the scene in terms of the system DoFs generically as Bx+d, and group all contact normals into a matrix U. Then, non-penetration constraints are mathematically formulated as

(3) $$C_{\mathrm{contact}}\left(x\right)=U^{T}\;\left(B\;x+d\right)=0.$$

Instead of enforcing these constraints exactly, we formulate a penalty energy. With a uniform stiffness *k* for all contacts, this penalty energy is:

(4) $$V_{\mathrm{contact}}\left(x\right)=\frac{1}{2}\;k\;C_{\mathrm{contact}}{\left(x\right)}^{T}\;C_{\mathrm{contact}}\left(x\right)=\frac{1}{2}\;k\;{\left(B\;x+d\right)}^{T}\;U\;U^{T}\;\left(B\;x+d\right).$$

This energy is simply added to the soft-tissue energy in (2).

#### 2.3.2. Tissue Fixing and Coupling

Soft tissue may be fixed in space or coupled to moving bones. We focus our discussion on tissue coupling, as tissue fixing can be regarded as a special case of coupling. Tissue coupling requires identifying a subvolume of the complete soft tissue, and then setting constraints on the corresponding nodes of the FEM discretization. While this is conceptually simple, our methods include two features that make the implementation highly efficient. First, during the simulation setup (see Section 2.5), we provide a simple interface to define couplings, leveraging a common meshing of the surfaces of soft tissue and bones. Second, we support this type of constraint in (8) without solving a complex constrained optimization, or without the need to assemble the Hessian of the soft tissue with a separation of coupled and free nodes.

We define a selection matrix S, which selects the coupled DoFs on the soft tissue. These constrained DoFs are defined as a linear function A of free DoFs z (which contain bone DoFs as defined in Section 2.2) and possibly some position offset c. In the case of fixed tissue, A=0, and c compiles the positions of fixed nodes, Then, we can define the coupling constraints as:

(5) $$C_{\mathrm{coupling}}\left(x\right)=S\;x-\left(A\;z+c\right)=0.$$

We also define explicitly the free DoFs as $z=\tilde{S}\;x$, where $\tilde{S}$ is another selection matrix, complementary to S, i.e., $\tilde{S}=I-S^{T}\;S$. We can reconstruct the full DoFs by combining the constrained and free DoFs as

(6) $$x={\tilde{S}}^{T}\;z+S^{T}\;A\;z+S^{T}\;c.$$

#### 2.3.3. Smooth Coupling at Bone Cuts

**Figure 2:**
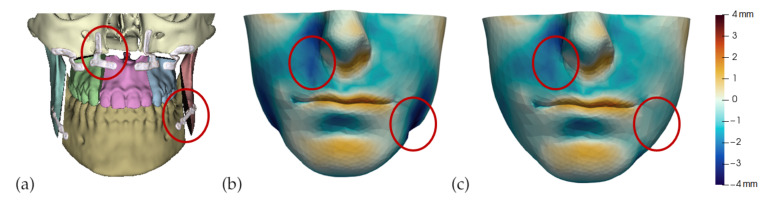
This figure shows the impact of the smooth coupling method described in Section 2.3.3 on the planning simulation for patient M2. (**a**) shows, color-coded, the different cuts applied to the maxilla and the mandible. It also shows implants added in the real surgery, with two of them highlighted with red ellipses. The next two images compare the planning simulation without smooth coupling (**b**) and with smooth coupling (**c**). With smooth coupling, the error is notably lower near the cuts, as shown for example in the two highlighted areas. Notice how the smooth coupling method even approximates the insertion of implants in a very easy yet accurate way.

We pay special attention to one particular type of coupling. When a bone is cut, adjacent portions of the soft tissue become coupled to different bone fragments. These fragments are transformed separately, and the adjacent portions of soft tissue may suffer unrealistically large local deformations. Implants are also often placed between fragments, as shown in Figure 2, which would further increase the local soft-tissue deformation.

We have designed a smooth coupling method for soft-tissue regions adjacent to bone cuts. This method builds on the technique of linear blend skinning (LBS) [41], which blends the rigid transformations of multiple bones. For each soft-tissue node, the LBS transformation can be expressed as a linear combination of rigid DoFs, hence it matches the general formulation of the coupling constraint (5). We define the weights of the bone transformations as follows. At the location of a bone cut, we use weights of 0.5 for the two resulting bone fragments. Then we linearly interpolate to weights of 1.0 and 0.0 along a distance of 1 cm from the cut.

Figure 2 compares the error on patient M2 with and without smooth coupling. As clearly visible in the areas highlighted with red ellipses, smooth coupling approximates in a simple yet accurate way the tissue deformation produced by cuts and also by the insertion of small implants. It removes the need for any complex modeling, and can be treated completely automatically within the modeling and simulation pipeline.

### 2.4. Constrained Optimization Problem

Next, we see how we leverage the definition of coupling constraints to efficiently solve a constrained optimization problem. Putting together the soft-tissue energy (2), the contact energy (4), and the coupling constraints (5), soft-tissue simulation can be formally posed as the constrained optimization

(7) $$x=\arg\min V_{\mathrm{soft}}\left(x\right)+V_{\mathrm{contact}}\left(x\right),\;\;\;\;s.t.\;C_{\mathrm{coupling}}\left(x\right)=0.$$

This optimization can be solved by performing Newton iterations. On each iteration the objective function is approximated quadratically using its Hessian $H=\frac{\partial^{2}V_{\mathrm{soft}}}{\partial x^{2}}+\frac{\partial^{2}V_{\mathrm{contact}}}{\partial x^{2}}$ and gradient $g={\frac{\partial V_{\mathrm{soft}}}{\partial x}}^{T}+{\frac{\partial V_{\mathrm{contact}}}{\partial x}}^{T}$ as

(8) $$x=\arg\min\frac{1}{2}\;x^{T}\;H\;x+g^{T}\;x,\;\;\;\;s.t.\;C_{\mathrm{coupling}}\left(x\right)=0.$$

However, thanks to the explicit transformation between free and full DoFs (6), we can directly express the optimization as an unconstrained optimization on the free DoFs:

(9) $$z=\arg\min\frac{1}{2}\;z^{T}\;\left(\tilde{S}+A^{T}\;S\right)\;H\;\left({\tilde{S}}^{T}+S^{T}\;A\right)\;z+{\left(g+H\;S^{T}\;c\right)}^{T}\;\left({\tilde{S}}^{T}+S^{T}\;A\right)\;z.$$

And the solution to this unconstrained optimization is trivially obtained by solving the following linear system:

(10) $$\left(\tilde{S}+A^{T}\;S\right)\;H\;\left({\tilde{S}}^{T}+S^{T}\;A\right)\;z=-\left(\tilde{S}+A^{T}\;S\right)\;\left(g+H\;S^{T}\;c\right).$$

As it becomes evident, with our approach to model constraints, the complex constrained optimization (8) becomes a simple linear system. The matrix of this linear system is built by first multiplying terms corresponding to the constrained DoFs by the matrix A, followed by a selection of rows and columns from the full Hessian. The right-hand side is built by applying the same procedure to the full gradient. After each linear-system solve, we execute a line search to guarantee that the total energy is reduced, and we continue with the next Newton iteration until the full simulation converges.

### 2.5. Preparation of Simulation Meshes and Couplings

**Figure 3:**
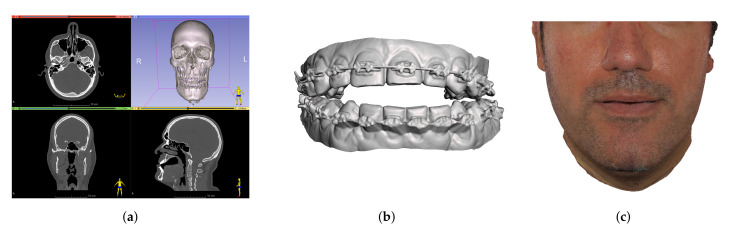
Summary of the patient imaging data collected for the planning process. CBCT-scan of the head (**a**), dental 3D scan (**b**), and textured face 3D scan (**c**) for patient M1.

In our computational planning approach, we assume the following patient imaging data is collected and input to the process (see Figure 3): a CBCT-scan, from which the different anatomical elements are segmented; a dental 3D scan, for high modeling and simulation accuracy of teeth; and a textured 3D scan of the face, which is only used for evaluation of results and is applied after the simulation. Setting up a simulation scene for pre-operative planning is a laborious task. Moreover, it often requires a combination of in-context knowledge from the anatomical, clinical, and simulation areas, to define simulation properties that are anatomically and clinically relevant. By approaching mathematical modeling and scene preparation in a cross-informed manner, we minimize the requirements on simulation knowledge for the technicians in charge of scene preparation.

The step of segmentation and meshing of the operation scene requires separating the volumetric anatomy into distinct objects, as well as defining interfaces between them. Our overall workflow is similar to the one followed by Mollemans et al [6]. However, in our approach the definition of object interfaces accounts for the models of boundary conditions described in Section 2.3, and is implemented through simple protocols. In this way, we are able to employ the simplified constrained optimization solver discussed in Section 2.1, which has a positive impact on the runtime cost of simulation during planning. To prepare the simulation data, we alternate volumetric and surface representations, which are best suited for different operations. For volumetric operations we leverage the open-source software 3D Slicer (v. 4.10.2, https://www.slicer.org/, accessed on 28 September 2021) [42], and for surface operations we leverage Meshmixer (v. 3.5, Autodesk Inc., Mill Valley, CA, USA).

#### 2.5.1. Bones

**Figure 4:**
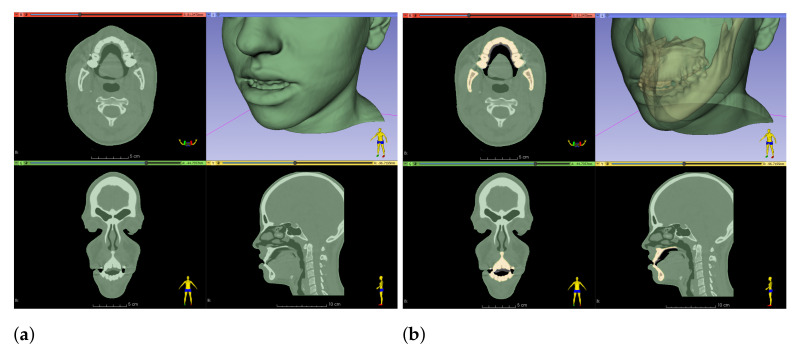
Results of segmentation in 3D Slicer. (**a**) shows segmentation of the entire patient. (**b**) shows segmentation of skin and bones.

Processing of bone structures includes three major steps: segmentation, cleanup, and preparation for cutting. We execute segmentation of the input CBCT-scan images on 3D Slicer. The images are first processed using a median filter, and then they are segmented using a thresholding algorithm. In this way, bone structures are separated from the soft tissue. Results of a segmentation in 3D Slicer are shown in Figure 4. Manual editing may be necessary in some areas like the mandibular condyles, and a smoothing filter is used for small regions.

We generate separate bone surfaces for the mandible and the skull, and we load them in Meshmixer for further clean-up. Meshmixer allows surface-editing operations to clean irregularities, close holes, remesh, simplify the mesh, make cuts, and many other options. First, surfaces are imported into the program, without any scale or rotation. Then, secondary meshes unintentionally generated during the segmentation process are discarded. The dental scan is also imported, registered and merged with the bone meshes through a Boolean union.

**Figure 5:**
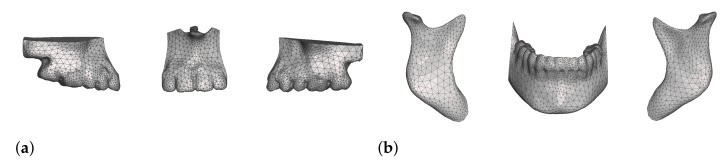
Meshes of bones after cleanup, cut and refinement in Meshmixer. Image (**a**) shows three maxillary segments; image (**b**) shows three mandibular segments.

The specification of cutting operations is a task to be performed by the clinician as a part of planning. We let the user place cutting planes relative to the bone meshes, and once verified we use them to separate the input meshes into two or more fragments. Each of these fragments can then be handled as a separate object in the simulation, and can be manipulated by the clinician during planning. In our project, we use Meshmixer to define and execute cutting operations. Figure 5 shows some examples of bone meshes after cleanup, cut and refinement in Meshmixer.

Once cuts are executed, all bone geometry is fully defined. At this point, we simplify bone meshes to reach just the necessary mesh complexity to represent the required detail, but without an excessive number of vertices which could slow down the planning simulation. Mesh quality must be checked and iterated if necessary. In Section 3, we extensively discuss how we manage to use meshes of low complexity, and hence high efficiency, without compromising planning quality. Once bone meshes are appropriately simplified, we pass them to the simulation engine for the definition of rigid-body DoFs as described in Section 2.2.

#### 2.5.2. Soft Tissue

Much of the process to prepare simulation meshes for soft tissue is the same as for bones. However, bone geometry is used as a reference to accurately find interfaces. The process starts with segmentation in 3D Slicer. In our experience, soft tissues require more manual intervention than bones, e.g., cleaning metal artifacts caused by braces, editing irregular areas like the lips, or applying smoothing filters to small regions. We also separate the lips in case the CBCT-scan was obtained with closed lips. Finally, we segment the air in the oral cavity using a simple thresholding approach, and we subtract it from the soft tissue using a Boolean difference operation.

**Figure 6:**
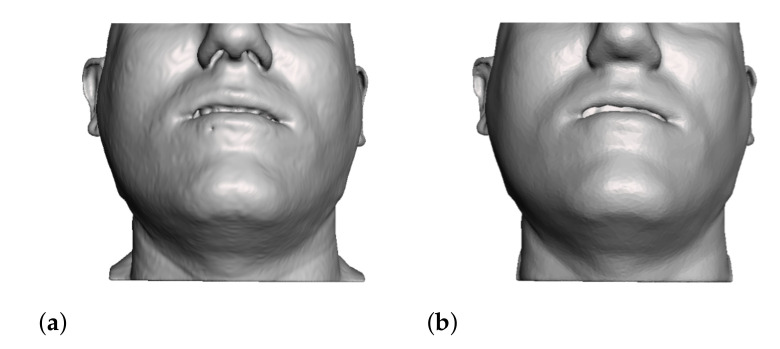
The preliminary segmentation of the skin mesh (**a**) is postprocessed to close small cavities and clean the surface (**b**).

We continue the cleaning process in Meshmixer. We close open areas of the soft tissues which are irrelevant from a clinical point of view, or which are far from the clinical region of interest, such as the openings of the respiratory tract (trachea and nostrils) and the external auditory canal (see Figure 6). We also discard small internal cavities. As a result of this cleaning and segmentation operation, we produce preliminary soft-tissue surfaces.

We then apply a Boolean difference operation between the preliminary soft-tissue surfaces and the bone surfaces. In particular, we apply this difference operation to the maxilla and the mandible. As a result of this operation, the surfaces of the tissue and the bones match exactly, and we leverage this newly created surfaces to simplify the definition of fixed tissue or tissue-bone couplings as described in Section 2.3.

**Figure 7:**
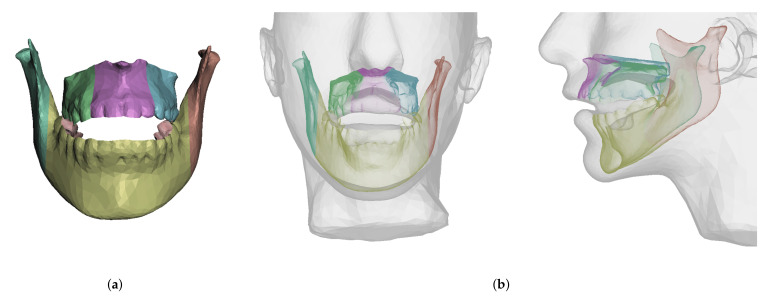
When cutting a bone, the couplings between this bone and soft tissue need to be accordingly split. We pseudo automatically separate the coupled surface of the soft tissue into different face groups in the Boolean subtraction operation. Image (**a**) shows bones cut into 6 fragments and image (**b**) shows the corresponding face groups in the interior of the skin mesh.

When the bones are cut as part of planning, the tissue-bone couplings must be migrated to the new bone fragments. To this end, we leverage the common meshing of coincident bone and tissue surfaces provided by the Boolean difference operation. If a bone is cut into multiple fragments, we identify the new surface fragments, and for each fragment we define a face group on the corresponding soft-tissue surface. Then, we set a tissue-bone coupling for each bone fragment and its corresponding soft-tissue face group. Figure 7 shows examples of bone fragments and coupled face groups on the soft tissue.

**Figure 8:**
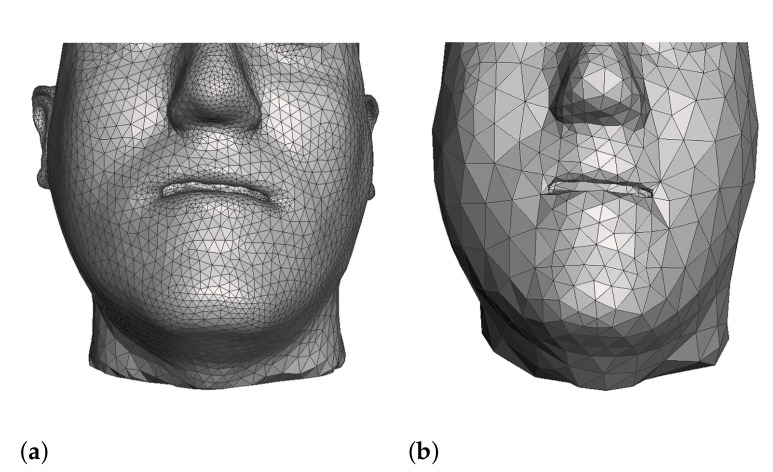
We simplify simulation meshes to retain the complexity that is sufficient for accurate planning, but which will minimize runtime cost. These images show examples where the raw mesh is simplified to 23,000 triangles (**a**) and 2600 triangles (**b**).

Last but not least, we execute a mesh simplification process to retain a mesh complexity that is sufficient for accurate planning, but which will minimize the runtime computational cost of the simulation, as shown in Figure 8. We use pseudoautomatic tools in Meshmixer to execute the simplification, requesting higher accuracy in regions of particular interest to the clinicians, such as the lips, the chin, or the nose. The result of this process is a set of soft-tissue surfaces, which are clean and closed. They are tetrahedralized using Tetgen, and the result defines the soft-tissue model as described in Section 2.1. The soft-tissue surfaces also enjoy well-defined interfaces with bone surfaces, which serve the definition of couplings as described in Section 2.3.

### 2.6. Textured Output Visualization

In this study, we focus on the quantitative evaluation of simulated planning. However, for effective presentation to the clinical team, it is important to visualize the planning results with the patient’s face texture.

**Figure 9:**
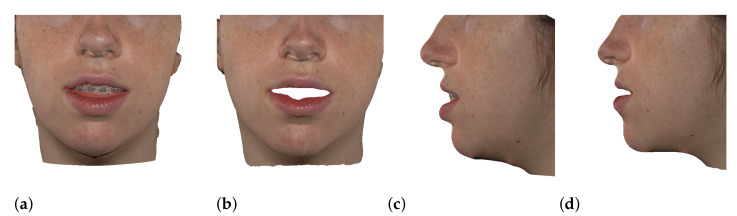
Preoperative 3D scan (**a**,**c**) and textured output (**b**,**d**) of patient M7, in frontal view (**a**,**b**) and lateral view (**c**,**d**).

To this end, we employ the pre-operative textured 3D scan of the patient’s face. After the generation of the high-resolution soft-tissue surfaces, we execute a rigid registration step to the textured scan. The result of this registration yields a mapping of the texture to the surface meshes, and we retain this mapping during mesh simplification. After every planning simulation, we simply render the simulation results with the overlay of the face texture (Figure 9).

## 3. Results

We have tested the described simulation methodology on a cohort of 10 orthognathic surgery patients. Informed consent for the use of medical images was obtained from all subjects, and the study was approved by the Ethics Committee of Hospital Universitario La Paz (Madrid, Spain).

In this section, we describe the validation methodology, we summarize the characteristics of the test cohort, we discuss the specific experiments that we have carried out, and we outline the results in terms of simulation error and simulation performance.

### 3.1. Validation Methodology

For each orthognathic patient analyzed in the study, we have collected post-operative CBCT-scans in addition to the pre-operative data outlined in Section 2.5. Based on the combination of pre- and post-operative data, we have identified the specific osteotomies and transformations applied to the patients, and we have carried out the same operations using our simulated planning methods. This enables a quantitative comparison of simulation results to ground-truth post-operative data.

**Figure 10:**
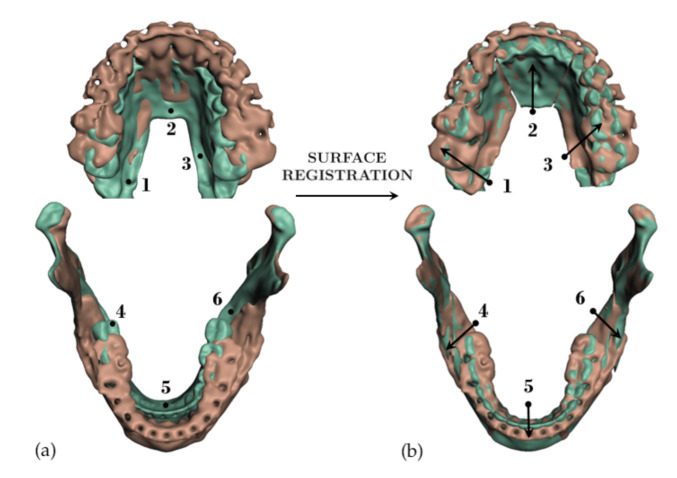
For validation of the proposed simulation methods, we simulate surgical procedures executed on actual interventions. To this end, we identify bone cuts in post-operative scans (brown), we apply those cuts to pre-operative scans (green), and we register the cut bone fragments between both scans. The images show the input pre-operative and post-operative scans (**a**), and again the same scans after registration (**b**). The transformations applied to the individual bone fragments (numbered 1 to 6 in the images) are used as boundary conditions for our planning simulation.

We start by segmenting bone geometry and bone fragments in the post-operative CBCT-scan, following the same procedure we describe for pre-operative data in Section 2.5. We identify the osteotomies executed to the maxilla and the mandible, and we apply the same cuts to the simulated bones. Next, we measure the transformations applied to the physical bone fragments, as these become the input to our simulation methods. We do this by executing a rigid registration operation between the pre- and post-operative fragments, using an Iterative Closest Point algorithm. Example results are shown in Figure 10.

Thanks to the registration results, we define the rigid transformations to be applied to the simulated bone fragments. Finally, with this information, we compute soft-tissue deformation by iteratively solving the numerical problem described in Section 2.4. To ensure robust computation of the deformations, we decompose the bone transformations in multiple substeps (10 in the examples), and for each substep we solve the nonlinear optimization (7) to convergence. This requires iterating the linear problem (10). Note that, on each iteration, the bone-tissue couplings extend the effect of the bone transformations to the soft tissue.

To measure error between the simulation results and the post-operative data, we load both data sets into 3D Slicer, and we use a signed closest-point distance computation. For this error analysis, we ignore regions outside the clinical region-of-interest, such as the neck and occipital areas, where the CBCT images tend to differ due to posture misalignment.

### 3.2. Test Cases

The test cohort of 10 patients has been selected in close collaboration with the Maxillofacial Surgery Service. The patients were selected to cover a large diversity of personal characteristics (e.g., diagnosis) and surgical procedures, as this diversity allows ample testing of the proposed simulation methodology. The main characteristics and the surgical procedures applied to all 10 patients are listed on Table 1.

We collected the following data for each patient: age (mean 32 years, range 22–51 years), gender (8 women, 2 men), ethnic group (8 Caucasian, 2 Latin American), and diagnosis (2 class II malocclusion cases, 4 class III malocclusion cases, 3 asymmetry cases, 1 open bite case). The surgeries undergone by the patients exhibit diverse procedures for both the maxilla and the mandible:

- In maxillary procedures, the maxilla is separated from the skull through a Lefort osteotomy, classified based on its anatomical level. In this cohort, the distribution of cases is: 8 Lefort I cases and 1 Lefort II case; one patient did not undergo maxillary surgery. Moreover, after a Lefort I osteotomy, the maxilla may be segmented (typically into three fragments) in order to expand the upper arch. Maxilla segmentation was applied to 6 patients in this cohort.
- In mandibular procedures, the mandible may be sagittally split on both rami (bilateral sagittal split osteotomy, BSSO) or only one ramus (unilateral sagittal split osteotomy, USSO). In this cohort, the distribution of cases is: 7 BSSO cases, 1 USSO case; two patients did not undergo mandibular surgery. Additionally, a chin osteotomy or genioplasty may be also performed. Genioplasty was applied to 1 patient in this cohort.

In total, 7 out of 10 patients underwent bimaxillary surgery, i.e., both maxillary and mandibular surgery. Table 2 and Table 3 show the pre-operative and post-operative scans for all 10 patients, as well as the bone fragments produced during surgery, before displacement and fixation.

The planning simulations have been performed with two different mesh resolutions for each patient. In this way, we compare accuracy and performance between fine and coarse simulations. Our hypothesis is that our modeling and simulation methodology, in particular the definition of couplings between anatomical elements, allows the use of coarse simulation meshes without incurring in excessive error. This would allow a large reduction of simulation times, even semi-interactive planning. Table 4 indicates the mesh complexity of both fine and coarse meshes for all patients. In all cases, the reduction in mesh complexity is between 80 and 90%.

### 3.3. Simulation Error and Performance

As described in Section 3.1, we evaluate signed distances between the simulation results and the post-operative scans in 3D Slicer. With this information, we compute color maps in Paraview v 5.8.1 (Kitware Inc., New York, NY, USA) [43] to visualize the error map. These error maps are visualized, per patient, in Table 2 and Table 3. The tables also compare the error maps using fine and coarse simulation meshes.

**Figure 11:**
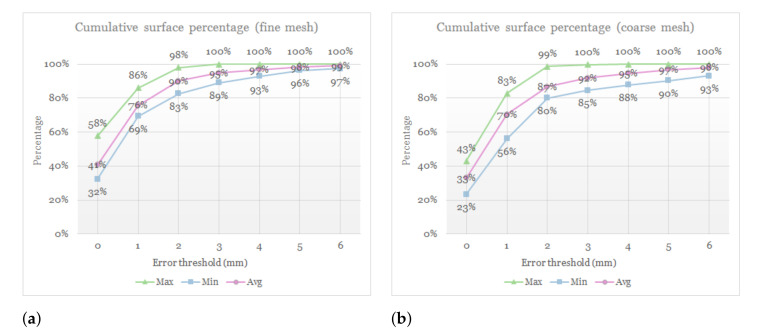
Plots of cumulative surface percentage with error below a predefined threshold. The plots show maximum, minimum, and average surface percentage across the 10 test patients. They also compare the results using a fine mesh (**a**) or a coarse mesh (**b**).

We have also computed, for each patient, the cumulative percentage of the surface with error below a predefined threshold. We have done this for several error thresholds spaced 1 mm. Figure 11 compares the cumulative surface percentage as a function of the error threshold, for fine and coarse meshes. The plots depict maximum, minimum, and average surface percentage across the 10 test patients. We validate that, for all the patients, the vast majority of the surface has an error lower than 3 mm, which is the clinically acceptable limit in orthognathic surgery planning [6,8,9,10,11,12,13]. Table 4 lists the cumulative surface percentage with an error lower than 3 mm for all the patients, comparing results with both fine and coarse meshes.

Finally, we have also measured the time required to compute the simulations on all patients. All simulations were executed on a commodity PC (Intel six-core i7 2.6 GHz CPU with 32GB RAM). Table 4 lists the computation times for all patients. With fine meshes, these times range between half a minute and over three minutes. With coarse meshes, on the other hand, they range between 3 and 15 s.

## 4. Discussion

The results outlined in the previous section can be analyzed from different angles. In this section, we analyze the overall accuracy of the planning simulation, but we also pay attention to possible differences across patients and clinical cases. Finally, we discuss the impact of the resolution of the simulation mesh.

### 4.1. Analysis of Simulation Accuracy

The error depicted in the form of color maps in Table 2 and Table 3 suggests that the error is dominated by negative values (i.e., cold colors) vs. positive values (i.e., warm colors). Negative error means that the simulation result appears inside the post-operative scan. The dominance of negative/cold error could be due to the following two factors, which should be studied in a future refinement of the simulation methodology.

First, during the preparation of the simulation meshes described in Section 2.5, the simulation mesh may shrink with respect to the original data set. This shrinking effect is a combined result of mesh cleaning, decimation, and refinement. Second, some elements of a real surgery are not simulated in our methodology, and this could also lead to a loss of volume in the final results. Such elements that are not simulated include bone grafts, bone fixation plates, or prostheses (see, e.g., patient M4).

The tendency toward negative/cold errors is slightly higher on coarse meshes. This is not a surprise, as mesh decimation is a possible source of shrinking as discussed above.

When analyzing the error on different regions of the face, we observe notable differences:

- Chin. Overall, the amount of error at the chin area is very low. This could be explained by the fact that the skin at the chin is very thin, and the coupling to the mandible makes the simulation highly predictive.
- Lips. In other regions, such as the lips, skin slides strongly over the underlying bones and teeth, and the deformation result is more difficult to predict. Overall, we observe higher variability in the error at the lips, and also some patients with higher error.
- Nose. The quality of the prediction of the deformation of the nose varies strongly across patients. In this case, the variability may depend on the type of surgery performed on each patient’s anterior nasal spine. This type of surgery is not easy to identify in the post-operative CBCT image due to the presence of bone grafts or fixation plates.
- Neck. Finally, we observe large error in the neck area (e.g., patients M5 and M8), and specifically at the junction point between the submental area and the neck (“C point” or “cervical point” in cephalometric analysis). This error was accounted for in our quantitative analysis, which negatively biased the overall results. However, this area is not of special interest to orthognathic surgeons. The deformation is known to be produced by a retraction of skin after surgery, but surgeons do not account for this effect during pre-operative planning.

### 4.2. Analysis of Clinical Cases and Patients

It is important to note that the error reported in the various tables and plots indicates absolute error, and does not take into account the amount of translation applied to the bone elements in the surgical intervention. Some cases require a large intervention and are therefore prone to higher error, such as the bimaxillary and segmented surgery of patient M1, while others require a milder intervention, such as the slight asymmetry of patient M9. These surgical differences translate into notable differences in the resulting absolute error, e.g., 92% and 100% of the surface with error below 3 mm on the coarse mesh for patients M1 and M9, respectively. Nevertheless, we opted to analyze absolute error as it is a better indicator of the clinical validity of the planning simulation.

At the same time, the diversity of surgical cases and personal characteristics of the patients allows us to carry out the following comparisons:

- Ethnicity. The predicted deformation of the central area of the face is visually more accurate for the Latin American patients M3 and M8) than for Caucasian patients (rest of patients). As discussed with collaborating surgeons, this may be due to stiffer soft tissue in the case of patients of Latin American ethnicity, which deforms in a more predictable way when bones are displaced, compared to Caucasian patients. However, the group of Latin American patients in the study is very small, and such ethnicity differences could be analyzed in a more thorough study.
- Diagnosis. Patients with Class II diagnosis exhibit distinct results with respect to the rest. In these patients (M5 and M6), the simulation result shows error in the deformation of the lower lip. Initially everted lips, such as those of these patients, do not reach the full deformation visible in the post-operative scans, where they appear in front of the teeth, but instead remain slightly everted. This simulation error may be caused by a lip stretching effect that is not correctly captured by the simulation model, and remains as one of the items to be improved in the future. Patients with Class III, asymmetry and open bite diagnoses do not exhibit any common error pattern within their groups.
- Lefort type. There appears to be a correlation between the type of Lefort osteotomy and the amount of error in the deformation of the nose. Specifically, the deformation of the nose is correctly predicted in the case of Lefort II osteotomy (patient M3), but it appears less predictable for patients with Lefort I osteotomy. This is probably due to the uncertainty of the intervention carried out on anterior nasal spine, as discussed earlier. Obviously, if a Lefort osteotomy is not performed (patient M9), there is no deformation and the prediction is correct.
- Segmentation of the maxilla and mandible. For all patients, the highest error (except for the neck, which is not clinically relevant as discussed above) appears near the cut areas, both of the maxilla (e.g., patients M5 and M7) and the mandible (e.g., patients M1 and M3). This is probably due to the presence of fixation plates and/or bone grafts in the real result (e.g., patient M10, whose maxilla was not segmented, but where the presence of bone graft has been confirmed by the surgeon who carried out the intervention). As a consequence, patients with a segmented maxilla and/or mandible show in general larger error than those without segmented bones. However, the smooth coupling method proposed in Section 2.3.3 reduces considerably the error in cut areas, as shown in Figure 2.
- Genioplasty. Error in the chin area appears low for patients who did not undergo genioplasty, but also for those who did (patient M4), as already mentioned. The analysis of genioplasty could be extended to a larger cohort.

### 4.3. Comparison of Fine and Coarse Meshes

The cumulative error analysis summarized in Figure 11 indicates a small loss of accuracy when the resolution of the simulation meshes is reduced. On average, 92% of the surface of the patients has an error lower than 3 mm with coarse meshes, and with fine meshes this percentage grows to 95%. Simulations with coarse meshes also exhibit a slightly wider range of error values.

However, when the specific patient cases are inspected in more detail, as depicted in Table 2 and Table 3, we can see that error appears in the same areas with coarse and fine meshes. The use of coarse meshes does not lead to additional sources of error, and the coarse and fine simulations are qualitatively equivalent.

The accuracy of coarse simulation meshes indicates that for most clinical cases they are perfectly valid, as the error in critical areas remains under clinically acceptable thresholds (i.e., 3 mm). In the worst case, the coarse simulation can be used as a faster preview of the clinical prediction, which can dramatically accelerate planning iterations. Only when the coarse simulation provides a clinically satisfactory result, surgeons may launch a fine simulation for higher accuracy.

The combination of coarse and fine simulations is further justified by the extreme reduction in computation times. As listed in Table 4, the reduction in simulation times achieved with coarse meshes (90.7% on average) is higher than the reduction in mesh complexity (84.8% on average). Moreover, this drastic reduction in simulation times produces only a minimal reduction in simulation accuracy (3.1% on average, measured as the cumulative surface percentage with error below 3 mm).

## 5. Conclusions

In this work, we have presented a simulation methodology for planning of orthognathic surgical interventions. The proposed methodology pays special attention to the definition of couplings between anatomical elements, e.g., bones and soft tissue. Complex handling of these couplings in previous work is a source of simulation complexity, which requires high-resolution meshes and long computation times to ensure accurate results. In contrast, the proposed methodology addresses in a combined manner the preparation of the simulation meshes and the computational definition of couplings, and results in runtime simulations that are accurate even with coarse meshes. The ability to use coarse meshes has a drastic impact on the simulation cost, as demonstrated in our results.

The analysis of results discussed in the previous section suggests that coarse meshes are accurate enough for full prediction of the clinical intervention in some cases. In other cases, due to the slight increase in error, we advise executing a final prediction using fine meshes. The use of coarse meshes can anyway have a strong impact in practical planning situations, as clinicians will be able to execute fast planning using coarse meshes as a good preview of the final result.

In this paper, we have carried out global quantitative validation of the simulation methodology. Further validation actions should include quantitative validation of anatomical landmarks, as well as qualitative clinical evaluation. For quantitative validation on landmarks, previous works [8] have proposed clinically interesting points, such as the tip of the nose, the lips, or the chin. For qualitative clinical evaluation, we will follow procedures carried out in previous work [6], with the help of the orthognathic surgery team at Hospital Universitario La Paz. An interesting possibility for further validation is to quantify errors in facial recognition, as explored by Olivetti et al. [44].

The proposed simulation methodology also admits further technical extensions. As discussed in Section 4.2, the lips appear to be the anatomical elements with higher modeling inaccuracies, in particular in case of everted lips. The soft-tissue model presented in Section 2.1 can be extended to include pretension, by defining rest configurations different than the one of the pre-operative scan.

Other areas of interest for future work include the acceleration of the model setup and the inclusion of richer planning functionalities. Currently, model setup requires multiple manual steps at both the volumetric and surface level. These steps could be accelerated with the use of model templates that are automatically morphed to patient data. Similarly, the cutting operations typically follow pre-defined clinical procedures, and they could be implemented as parametric operations that are easily applied on the patient’s model. Finally, the textured visualization of the simulation results, described in Section 2.6, could be leveraged during planning operations and/or for communication purpose.

## Figures and Tables

**Table 1:** Characteristics of the 10 patients analyzed in the study, including surgical procedures applied to maxilla and mandible.

ID	Gender	Age	Ethnic Group	Diagnosis	Maxilla Surgery		Mandible Surgery	
					Lefort Type	Segmented	Sagittal Split	Genioplasty
M1	M	41	Caucasian	Class III	I	Yes	BSSO	No
M2	F	31	Caucasian	Open bite	I	Yes	BSSO	No
M3	F	36	Latin American	Class III	II	No	BSSO	No
M4	F	28	Caucasian	Asymmetry	I	No	USSO	Yes
M5	F	25	Caucasian	Class II	I	Yes	BSSO	No
M6	M	51	Caucasian	Class II	I	Yes	BSSO	No
M7	F	22	Caucasian	Class III	I	Yes	No	No
M8	F	22	Latin American	Class III	I	Yes	No	No
M9	F	36	Caucasian	Asymmetry	No	No	BSSO	No
M10	F	29	Caucasian	Asymmetry	I	No	BSSO	No

**Table 2:** Simulation results for patients M1 to M5. From left to right: pre-operative scan, post-operative scan, bone fragments produced during surgery, simulation error using a fine mesh, and simulation error using a coarse mesh. Scale of the color maps ranges from −4 mm to 4 mm.

ID	Skin Pre	Skin Post	Bones	Fine Mesh Error	Coarse Mesh Error	
M1	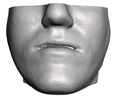	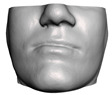	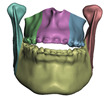	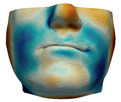	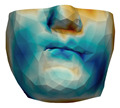	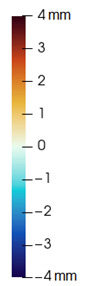
M2	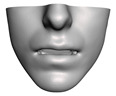	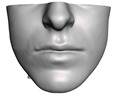	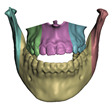	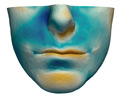	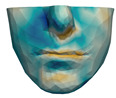	
M3	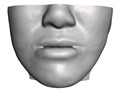	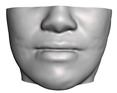	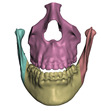	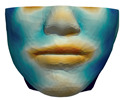	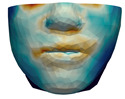	
M4	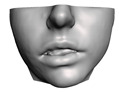	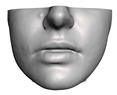	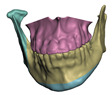	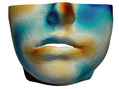	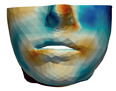	
M5	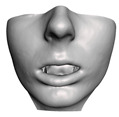	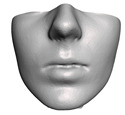	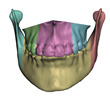	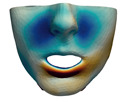	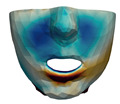	

**Table 3:** Simulation results for patients M6 to M10. From left to right: pre-operative scan, post-operative scan, bone fragments produced during surgery, simulation error using a fine mesh, and simulation error using a coarse mesh. Scale of the color maps ranges from −4 mm to 4 mm.

ID	Skin Pre	Skin Post	Bones	Fine Mesh Error	Coarse Mesh Error	
M6	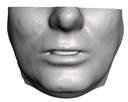	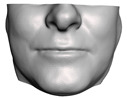	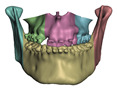	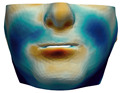	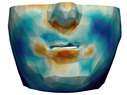	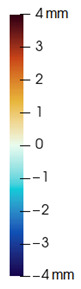
M7	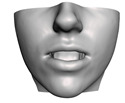	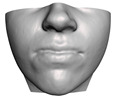	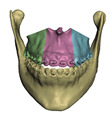	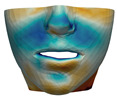	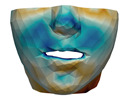	
M8	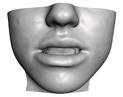	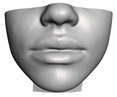	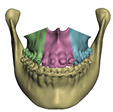	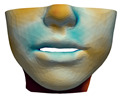	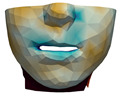	
M9	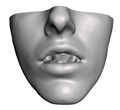	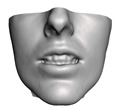	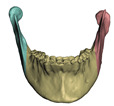	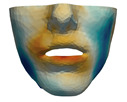	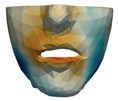	
M10	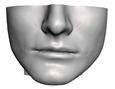	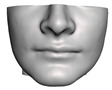	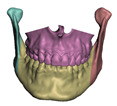	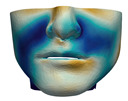	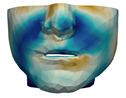	

**Table 4:** Summary of simulation results for all patients. The table compares simulation time and error for fine and coarse meshes.

Patient ID	Number of Triangles			Simulation Time (s)			Surface with Error <= 3 mm		
	Fine	Coarse	Reduction	Fine	Coarse	Reduction	Fine	Coarse	Reduction
M1	22,970	2600	89%	90.7	5.7	93%	98%	92%	6%
M2	18,000	3400	81%	111.6	11.8	90%	98%	96%	2%
M3	18,600	3800	80%	68.5	12.6	82%	95%	90%	5%
M4	22,750	4250	81%	107.3	14.9	86%	95%	93%	2%
M5	19,500	3848	80%	202.7	11.8	94%	89%	85%	4%
M6	22,560	2720	88%	102.4	10.7	92%	94%	91%	3%
M7	23,576	2632	89%	111.9	4.8	96%	91%	91%	0%
M8	22,888	2354	90%	77.5	3.8	95%	93%	86%	7%
M9	18,738	2646	86%	38.6	3.3	92%	100%	100%	0%
M10	20,640	3390	84%	65.9	9.3	87%	96%	94%	2%

## Data Availability

Project data is available under request from the authors.

## Sample Availability

No samples are available for this project.
